# Advancing Adult Day Services Research: The 2016 and 2018 National Study of Long-Term Care Providers

**DOI:** 10.1093/geroni/igaa057.2100

**Published:** 2020-12-16

**Authors:** Jessica Lendon, Vincent Rome, Christine Caffrey, Priyanka Singh, Manisha Sengupta

**Affiliations:** 1 National Center for Health Statistics, Hyattsville, Maryland, United States; 2 Centers for Disease Control and Prevention/National Center for Health Statistics, Hyattsville, Maryland, United States

## Abstract

This presentation demonstrates how researchers can leverage data from the 2018 redesign and new content from the forthcoming NSLTCP survey of adult day services centers (ADSC) conducted by National Center for Health Statistics. For the first time, NSLTCP data will allow analyses at the services-user level. New policy-relevant topics about centers and ADSC participants include reasons for hospitalization, medication use, patient-centered and end-of-life care, staffing turnover, and unmet needs. Additionally, the presentation highlights latest findings from the 2016 survey. About 53% of centers were primarily medical model. Almost 79% of participants in medical model centers used Medicaid, compared to 51% in social model centers. About 4% of participants had at least one 90-day hospitalization. 40% of participants had difficulty bathing. The most prevalent chronic conditions were hypertension (50%), arthritis (38%), and diabetes (31%). Nearly 40% of participants had an advance directive. Findings are contextualized within the broader understanding of ADSCs.

